# Adjunctive Therapy Approaches for Ischemic Stroke: Innovations to Expand Time Window of Treatment

**DOI:** 10.3390/ijms18122756

**Published:** 2017-12-19

**Authors:** Talia Knecht, Jacob Story, Jeffrey Liu, Willie Davis, Cesar V. Borlongan, Ike C. dela Peña

**Affiliations:** 1 Department of Pharmaceutical and Administrative Sciences, Loma Linda University School of Pharmacy, Loma Linda, CA 92350, USA; knecht.talia@gmail.com (T.K.); jstory512@gmail.com (J.S.); jefliu@llu.edu (J.L.); wldavis@llu.edu (W.D.); 2 Department of Psychology, University of California, San Diego, CA 92093, USA; 3 Department of Neuroscience, University of California, Riverside, CA 92521, USA; 4 Department of Neurosurgery and Brain Repair, Center of Excellence for Aging and Brain Repair, University of South Florida College of Medicine, Tampa, FL 33612, USA; cborlong@health.usf.edu

**Keywords:** tissue plasminogen activator, hemorrhage, blood-brain barrier, stem cell, matrix metalloproteinase (MMP)

## Abstract

Tissue plasminogen activator (tPA) thrombolysis remains the gold standard treatment for ischemic stroke. A time-constrained therapeutic window, with the drug to be given within 4.5 h after stroke onset, and lethal side effects associated with delayed treatment, most notably hemorrhagic transformation (HT), limit the clinical use of tPA. Co-administering tPA with other agents, including drug or non-drug interventions, has been proposed as a practical strategy to address the limitations of tPA. Here, we discuss the pharmacological and non-drug approaches that were examined to mitigate the complications—especially HT—associated with delayed tPA treatment. The pharmacological treatments include those that preserve the blood-brain barrier (e.g., atovarstatin, batimastat, candesartan, cilostazol, fasudil, minocycline, etc.), enhance vascularization and protect the cerebrovasculature (e.g., coumarin derivate IMM-H004 and granulocyte-colony stimulating factor (G-CSF)), and exert their effects through other modes of action (e.g., oxygen transporters, ascorbic acid, etc.). The non-drug approaches include stem cell treatments and gas therapy with multi-pronged biological effects. Co-administering tPA with the abovementioned therapies showed promise in attenuating delayed tPA-induced side effects and stroke-induced neurological and behavioral deficits. Thus, adjunctive treatment approach is an innovative therapeutic modality that can address the limitations of tPA treatment and potentially expand the time window for ischemic stroke therapy.

## 1. Introduction

Stroke persists as one of the most prolific killers of Americans, and poses a considerable threat to millions of others worldwide [1]. The therapeutic options for this disease are limited, and most of the currently used medications show limited efficacy in restoring lost neurological functions. Furthermore, there is but one Food and Drug Administration (FDA)-approved drug for stroke, namely, tissue plasminogen activator (tPA), which presents significant limitations: a time-constrained therapeutic window (the drug must be given within 4.5 h from stroke onset), and adverse side effects associated with delayed treatment of the drug, most notably hemorrhagic transformation (HT) [2]. These hurdles of tPA treatment result in a mere 3 percent of ischemic stroke patients actually benefiting from tPA therapy [3,4,5]. As a result of the scarcity of effective therapies and other unmet clinical needs for stroke, preclinical and clinical research for novel stroke interventions have been initiated.

An assortment of drugs ranging from those that augment neurogenesis [6] and other thrombolytic agents [7,8] have been tested with poor clinical results. As reperfusion with tPA continues to be regarded as the gold standard treatment for ischemic stroke, a considerable clinical dilemma at hand is identifying strategies that will enhance the therapeutic time window for tPA therapy and curtail the adverse effects (especially HT) of tPA treatment [9]. Therefore, identifying interventions that will address the aforementioned impediments of tPA therapy is as important as developing new drugs for acute ischemic stroke [9]. Expanding the thrombolytic time window for ischemic stroke treatment via combination therapy will not only minimize the complications or detrimental side effects of delayed tPA treatment, but also allow the time window of neuroplasticity to remain open for a longer period of time, likely resulting in improved recovery and functional outcomes post-treatment.

## 2. Adjunctive Treatment to Expand Therapeutic Time Window for tPA

**Figure 1:**
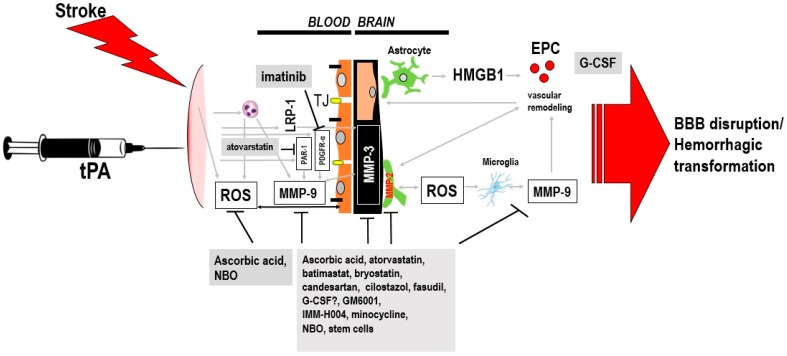
Proposed molecular targets of adjunctive treatments to enhance therapeutic window of tissue plasminogen activator (tPA) treatment. Acute stroke may cause injury to endothelial cells causing release of free radicals and pro-inflammatory cytokines. The signaling actions of tPA on the neurovascular unit may also increase blood-brain barrier (BBB) leakage, neurovascular cell death, and hemorrhagic transformation (HT). Moreover, the HT that ensues after delayed tPA treatment has been attributed to increased reperfusion and the effect of tPA on metalloproteinase (MMP) activity and other signaling pathways, including lipoprotein receptor-related protein (LRP), protease-activated receptor (PAR1), and PDGRF-α signaling. Ascorbic acid, normobaric oxygen (NBO) attenuates delayed tPA-induced complications in preclinical stroke models via inhibition of ROS production and BBB protection. Atovarstatin, minocycline, cilostazol, GM6001, fasudil, candesartan, bryostatin, and IMM-H004 reduces the HT by preserving the BBB through their actions on various MMPs and tight junction proteins. Granulocyte-colony stimulating factor (G-CSF) and IMM-H004 may reduce the HT by enhancing neurovascularization in addition to restoring BBB integrity. Imatinib reduces HT through the PDGRF-α receptor, while atovarstatin exerts its therapeutic benefits via inhibition of PAR1. Stem cells may also exert multi-pronged effects including BBB protection via its actions on various matrix metalloproteinases (MMPs). Abbreviations: EPC, endothelial progenitor cell; G-CSF, granulocyte-colony stimulating factor; HMGB1, high-mobility-group-box-1; ROS, reactive oxygen species; LRP, lipoprotein receptor-related protein; PAR1, protease-activated receptor; PDGFR-α, platelet-derived growth factor α-receptor (PDGFR-α); NBO, normobaric oxygen.

Disruption of the blood-brain barrier (BBB), damage to microvessels, and the toxic and non-thrombolytic actions of tPA have been suggested as the mechanisms underlying delayed tPA-induced complications, especially HT [8,10,11,12,13]. Pharmacological and non-drug interventions that counter the above events and target the molecules that contribute to BBB disruption, promote vascularization, etc., are logical treatments that could be given along with tPA to prevent such complications. Moreover, treatments with multi-pronged therapeutic effects are ideal in view of the complex mechanisms of stroke and delayed tPA-induced HT [9,14]. In the following sections, we discuss the pharmacological and non-drug treatments that have been examined to attenuate the complications, especially HT, of delayed tPA treatment. We focus on interventions that have been tested in experimental animal models, whereby delayed tPA treatment has been defined as >4.5 h after stroke onset. When available, data describing the performance of these agents in clinical studies are also discussed. These adjunctive treatments, their effects, and proposed mechanisms of action are shown in Figure 1 and summarized in Table 1 and Table 2.

## 3. Pharmacological Approaches to Extend Thrombolytic Time Window for Ischemic Stroke Treatment

The HT following delayed tPA treatment can be curtailed by an intervention that could help preserve the integrity of the BBB. Of note, stabilizing the BBB after stroke has been suggested to enhance the overall efficacy of tPA reperfusion therapy [11]. In light of the participation of metalloproteinases (MMPs) in the disruption of the BBB [8,10,11,12,13], targeting various MMPs has been explored. Moreover, preserving endothelial tight junction proteins (TJP) has also been considered, given that TJPs comprise the basic structure of the BBB [21,29,30]. Examples of pharmacological agents that exert therapeutic benefits by preserving the BBB are atovarstatin, batimastat, bryostatin, candesartan, cilostazol, fasudil, minocycline, etc. Vascular disruption plays a key role in intracerebral hemorrhage resulting in BBB leakage [31]. Thus, in addition to restoring BBB integrity, enhancing neovascularization or blood vessel formation is a logical strategy to counteract delayed tPA-induced HT. Angiogenesis, or the formation of new blood vessels, is also initiated in the ischemic region post vascular occlusion and contributes to improvements following infarction and neuronal recovery [32]. The pharmacological agents investigated to attenuate side effects of delayed tPA treatment by enhancing vascularization and protecting the cerebrovasculature include the coumarin derivative IMM-H004 and granulocyte-colony stimulating factor (G-CSF). Also, considering the role of free radicals in the complications associated with delayed tPA treatment, the effects of antioxidants have also been investigated [15]. The potential of oxygen transporters, which are promising stroke treatments based on preclinical studies, has also been recently explored for their ability to enhance the therapeutic window of tPA [20].

### 3.1. Ascorbic Acid

That glutathione and ascorbic acid (AA) levels decrease and free radical formation increases after ischemic brain injury (IBI) indicate the potential of AA supplementation to improve outcomes after IBI [33]. Ascorbic acid, or vitamin C, which could preserve endothelial function against ischemic oxidative injury in diabetes and counteract the formation of free radicals in the brain parenchyma, may attenuate the adverse effects of delayed tPA treatment [15]. In rats subjected to permanent middle cerebral artery occlusion (MCAO) and administered with low dose tPA (1 mg/kg, intravenous (i.v.)) and oral vitamin C (500 mg/kg) at 5 h after stroke, infarct volume and edema were reduced at 48 h post stroke, in comparison with rats given tPA only [15]. MMP-9 formation is triggered by oxidative stress which, in turn, promotes BBB damage after ischemia-reperfusion. The increase in MMP-9 levels and BBB disruption due to delayed tPA treatment were reduced by vitamin C administration [15]. Thus, vitamin C supplementation attenuates some of the deleterious side effects of delayed tPA therapy and exerts neuroprotection, indicating its potential as an adjunctive treatment to expand the limited therapeutic window of tPA. While vitamin C supplementation has been shown to improve stroke volumes, its impact on HT has not yet been determined.

### 3.2. Atorvastatin

The pleiotropic (e.g., antithrombotic, anti-inflammatory, and BBB-preserving) effects of statins make them attractive co-treatments to reduce the complications of delayed tPA treatment, as well as to extend the therapeutic window of the drug [16]. Atorvastatin, administered at 4 h after embolic stroke in rats, was found to attenuate the embolus size at the origin of the middle cerebral artery, improve microvascular patency, and decrease infarct volume in animals treated with tPA at 6 h after stroke. Moreover, the combination therapy did not increase the incidence of HT. The tPA-induced increase of protease-activated receptor-1, intercellular adhesion molecule-1, and MMP-9 were decreased by atovarstatin. Atovarstatin also reduced cerebral microvascular platelet, neutrophil, and fibrin deposition. It has been proposed that atorvastatin-induced reduction of delayed tPA-potentiated adverse cerebrovascular events contributed to the neuroprotective effect of the drug [16]. The latter has been attributed to the thrombolytic efficacy of atovarstatin, which leads to enhanced cerebrovascular patency and integrity [16].

### 3.3. Batimastat (BB-94)

Batimastat is a broad-spectrum MMP inhibitor [17]. Treatment with batamistat (50 mg/kg, intraperitoneal (i.p.)), in spontaneously hypertensive rats subjected to embolic stroke was shown to significantly reduce the volume of delayed tPA (6 h post stroke)-associated cerebral hemorrhage [17]. However, the specific MMP members and pathways involved in the therapeutic effect of batimastat were not explored. Moreover, despite the reduction in hemorrhage, no remarkable attenuation of neurological deficits post stroke was observed in batimastat-treated animals. Time- and dose-response studies are warranted to determine the optimal treatment regimen of batimastat with tPA in experimental stroke models.

### 3.4. Bryostatin

The efficacy of the protein kinase C (PKC) modulator bryostatin (2.5 mg/kg; i.v.), given 2 h post MCAO to reduce delayed tPA (5 mg/kg, i.v.)-induced cerebral swelling, hemorrhage, and mortality at 24 h post MCAO in rats was investigated [18]. Notably, bryostatin decreased ischemic brain injury in aged female rats [34]. In rats subjected to delayed tPA treatment, bryostatin attenuated the HT and BBB disruption, and decreased MMP-9 expression while upregulating PKCε expression [18]. The bryostatin-mediated decrease in MMP-9 has been suggested to produce outcome improvements post-stroke. Moreover, bryostatin-induced upregulation of PKCε was also hypothesized to decrease damage to TJPs within the BBB and reduce the HT [18]. PKCε regulation of MMP-9 was also proposed to play an important role in the beneficial effect of bryostatin to reduce delayed tPA-induced hemorrhage and BBB disruption [18]. 

### 3.5. Candesartan

Candesartan blocks the angiotensin II type 1 receptor and prevents injury due to ischemic stroke [19]. Early treatment with candesartan (1 mg/kg, at 3 h after stroke onset) has been shown to decrease the brain hemorrhage and improve neurological outcomes in animals subjected to embolic strokes and given tPA (10 mg/kg, i.v.) at 6 h after stroke [19]. However, the combination therapy increased MMP-9 levels although it decreased MMP-3 levels. The intracranial bleeding after tPA treatment in stroked mice was also decreased in MMP-3-null, but not MMP-9-null mice compared to wild-type controls [35]. In view of the above findings, it was proposed that activation of MMP-9 alone is not enough to increase the incidence of hemorrhage in embolic stroke. Nevertheless, the combination therapy decreased nuclear factor kappa-B (NF-κB) expression, which has been shown to mediate MMP-3 expression in endothelial cells after tPA treatment, and also to decrease TNF-α expression following activation of NF-κB. Subjects given candesartan also showed enhancement in the activation of endothelial nitric oxide synthase, an enzyme required for vascular function and homeostasis [36].

### 3.6. Cilostazol

Cilostazol is used for the treatment of intermittent claudication [37]. Combination treatment with cilostazol (10 mg/kg; i.v.) and tPA (10 mg/kg, i.v.) at 6 h post stroke after reperfusion in mice has been shown to reduce HT, brain edema, morbidity and mortality, and neurological deficits at 18 h and 7 days after the reperfusion [30]. Cilostazol treatment also attenuated delayed tPA-induced upregulation of MMP-9 activity and counteracted the decrease in expression of claudin-5 [30], an essential molecule for the assembly of tight junctions between microvascular endothelial cells [38]. In vitro, cilostazol prevented the tPA-induced damage on endothelial cells and pericytes via its effects on cyclic adenosine monophosphate (cAMP) activity [30]. It remains to be known whether the neurovascular protective effects of the cilostazol persist for longer time periods post-stroke.

### 3.7. Dodecafluoropentane Emulsion (DDFPe) Nanodroplets

Dodecafluoropentane emulsion (DDFPe) is an oxygen-transporting perfluorocarbon given i.v. shown to provide neuroprotection in rabbits subjected to ischemic stroke [20]. The efficacy of DDFPe (0.3 mL/kg) to enhance the time window for tPA treatment was examined in rabbits which underwent embolic stroke procedures [20]. DDFPe treatment has been shown to reduce the neurological deficits and stroke volumes at 24 h post stroke in rabbits given tPA (0.9 mg/kg, given 9 h after stroke) [20]. Improved oxygen transport without the need for red blood cell flow has been proposed as the mechanism underlying the therapeutic efficacy of DDFPe [20]. The impact of DDFPe treatment on HT associated with delayed tPA administration has not yet been studied.

### 3.8. Fasudil

Fasudil has been marketed in Japan for the treatment of cerebral vasospasms occurring after subarachnoid hemorrhage [39]. It is a Rho kinase inhibitor initially described as an intracellular calcium antagonist. Fasudil (3 mg/kg, i.p.) has been shown to decrease HT at 18 h post reperfusion in mice subjected to 6-h MCAO and treated with tPA (10 mg/kg, i.v.) [21]. It also remarkably decreased mortality and improved locomotor activity in stroked animals at 7 days after the reperfusion. Fasudil treatment, however, did not exert neuroprotection when compared with controls and tPA-alone treatment group [21]. The in vitro studies showed that fasudil prevented the tPA-induced injury to human brain microvascular endothelial cells (HBMECs) via reduction of MMP-9 activity [21]. The lactate dehydrogenase assays also revealed that fasudil prevented tPA-induced damage by protecting the endothelial cells [21]. Exploring the long-term neurovascular protective effects of fasudil, the molecular mechanisms in delayed tPA-induced HT, and also the optimum doses of the drug when combined with tPA are worthwhile future research endeavors [21].

### 3.9. Granulocyte Colony-Stimulating Factor (G-CSF) 

Granulocyte-colony stimulating factor (G-CSF) is an FDA-approved medical countermeasure to promote survival in patients exposed to myelosuppressive doses of radiation. Functionally, it is a cytokine which regulates the survival, proliferation, and differentiation of hematopoietic stem cells and hematopoietic progenitor cells [40]. G-CSF (300 μg/kg, i.v.) treatment has been shown to reduce delayed (6 h post MCAO) tPA (10 mg/kg, i.v.)-induced HT [22]. It also increased levels of angiogenesis marker Ang-2 but not Ang-1, vasculogenesis marker vWF, phosphorylated-eNOS, and endothelial progenitor cell (EPC) markers cluster of differentiation (CD) 34+ and vascular endothelial growth factor receptor (VEGFR)-2 in the ischemic hemispheres of stroked rats compared with rats given tPA treatment only. The neurological deficits at 24 h post drug treatment were also improved by G-CSF treatment. It has been proposed that G-CSF reduces delayed tPA-induced HT and enhances the neurological outcomes post stroke via angiogenic and vasculogenic activities of G-CSF, proliferative or regenerative actions of G-CSF-recruited EPCs, or both [22]. Although completion of vascularization typically requires several days, drugs that promote vascularization in stroke may accelerate the process and promote preservation of a patent vasculature against tPA-induced HT. Notably, a clinical study found that while the growth factors (GFs) vascular endothelial growth factor (VEGF), Ang-1 and G-CSF enhanced recanalization; Ang-1 but not VEGF or G-CSF enhanced HT [41]. High serum levels of G-CSF correlated with improved functional outcomes even at 90 days post treatment [41]. These results highlight the potential of G-CSF to reduce delayed tPA treatment-associated complications.

### 3.10. Ilomastat (GM6001)

GM6001 attaches to the active sites of MMPs and prevents the conversion of pro-MMPs to active forms of matrix-degrading MMPs [42]. GM6001 (100 mg/kg, i.p.) treatment in mice subjected to filamental MCAO and delayed tPA (10 mg/kg, i.v.) therapy (6 h post stroke) remarkably decreased tPA-induced elevation in brain hemoglobin, indicating that the drug reduced delayed tPA-associated HT [23]. GM6001 treatment also reduced tPA-elevated MMP-9 at 42 h after the reperfusion, and the degradation of occludin and ZO-1 but not claudin-5 expression [23]. Moreover, GM6001 also increased the survival rate and the reduction in locomotor activity in animals at 7 days after ischemia and reperfusion [23]. In vitro studies showed that GM6001 countered tPA-induced damage in endothelial cells and the decrease in transendothelial electrical resistance [23]. Considering that GM6001 inhibited tumor necrosis factor-α (TNF-α) converting enzyme (TACE) expression and that increased levels of TNF-α correlates with intracerebral hemorrhage in animal models [43], the interaction between GM6001 and these molecules needs to be explored.

### 3.11. Imatinib

Imatinib is a platelet-derived growth factor α-receptor (PDGFR-α) inhibitor approved by the FDA for the treatment of chronic myelogenous leukemia and other cancers. The drug, given orally at a high dose of 200 mg/kg, at 1 h after ischemia via photothrombosis, was observed to reduce the extent of HT after delayed (5 h post stroke) treatment with tPA [24]. It also reduced the cerebrovascular permeability and stroke lesion volume. As it was given at 1 h post stroke, it remains to be known whether it is also effective in reducing complications associated with delayed tPA treatment when given at later time-points after stroke [24].

### 3.12. IMM-H004, a Coumarin Derivative

IMM-H004 is a coumarin derivative which belongs to a class of organic heterocyclic compounds with numerous biological effects [44]. In rats subjected to embolic stroke and given tPA (10 mg/kg, i.v., 6 h post stroke), IMM-H004 (6 mg/kg, i.v.) treatment decreased the hemorrhage, infarction volume, and cerebral edema [25]. IMM-H004 also reduced tPA-mediated HT and enhancement of ischemic infarction in rats subjected to stroke via the intraluminal filament method. Decreasing MMP-9/MMP-2, promoting co-localization of MMP-2 with astrocytes and IgG leakage, and increasing occludin levels were the mechanisms proposed to underlie the efficacy of IMM-H004. Moreover, IMM-H004 promoted vascularization and increased cerebral perfusion at 7 days post stroke by improving the integrity of vascular endothelial cells. The in vitro studies revealed that IMM-H004 increased levels of ATP and the protein kinase A (PKA) and PI3K-dependent activation of Akt in HBMECs and PC12 cells, suggesting the involvement of cAMP/PKA and PI3K/Akt signaling pathways [25]. Thus, IMM-H004 may attenuate delayed tPA-induced HT by enhancing neurovascularization along with preventing BBB disruption [25].

### 3.13. Minocycline

Clinically used for the treatment of acne vulgaris, minocycline (3 mg/kg, i.v., at 4 h post stroke) has been shown to reduce infarction and attenuate the brain hemorrhage observed 24 h after embolic stroke [26] in animals treated with tPA (10 mg/kg, i.v., at 6 h post stroke). As a potent MMP inhibitor [45], minocycline decreased plasma MMP-9 levels which coincided with volumes of infarction and hemorrhage [26]. It remains to be known whether brain MMP-9 levels are also reduced by minocycline and whether MMP-9 levels correlate with the extent of infarction and hemorrhage [26]. In an exploratory trial to measure safety and efficacy of minocycline when given in combination with tPA [46], 60% of patients given a loading dose of minocycline within a 6-h time window followed by maintenance dosing for 3 days showed no incidence of intracerebral hemorrhage. Subjects given tPA in the minocycline trial also showed lower plasma MMP-9 levels [47]. Other clinical trials in different populations have been started and are awaiting results [48].

## 4. Non-Drug Adjuvants to Extend Thrombolytic Time Window for Ischemic Stroke Treatment

Expanding the time window for thrombolysis may not only be achieved through pharmacological means, but also through non-drug strategies [9]. The multi-pronged effects of stem cells indicate their worth as treatments to attenuate the complications associated with delayed tPA treatment [9,49,50]. Gas therapy, which has been considered as a logical ischemic stroke treatment, has also been examined for its potential application to counter delayed tPA treatment-associated outcomes [28]. Other non-drug strategies are well-described techniques, such as brain imaging and endovascular procedures, that have been shown clinically to visualize stroke pathology and treatment efficacy, as well as to help extend the therapeutic window for tPA treatment in ischemic stroke [9,51,52,53].

### 4.1. Minocycline and Neural Stem Cells

Minocycline has been previously shown to reduce hemorrhage associated with delayed tPA treatment [17]. Intracranial transplantation of neural stem cells (hNSCs) has also been demonstrated to mitigate the BBB damage caused by ischemic stroke [54]. In mice subjected to MCAO followed by reperfusion and given tPA at 6 h post stroke, minocycline reduced the mortality associated with delayed tPA treatment, especially in aged mice [27]. Moreover, significant attenuation of delayed tPA-induced pathophysiology was observed in mice treated with minocycline and intracranially transplanted with hNSCs at 24 h post stroke [27]. Thus, the combination therapy of tPA and minocycline, and stem cell transplantation could not only mitigate delayed tPA-induced side effects, but also enhance neuroplasticity post stroke. 

Other types of stem cells have also been investigated and have shown promise in mitigating the complications associated with tPA treatment. Mesenchymal stem cells (MSCs) which have been shown to reduce stroke volume and behavioral deficits in stroke models (for review, [55]), also reduced incidence of hemorrhage and improved behavioral dysfunctions in rats subjected to tPA (1 h 30 min post stroke, after reperfusion) treatment [56]. The treatment also reduced MMP-9 levels in the combination tPA + MSC group, compared with tPA alone-treated subjects [56]. MSCs may inhibit endothelial dysfunction to suppress hemorrhagic events and facilitate functional outcome. The combination MSCs and tPA therapy may also produce early behavioral recovery. Bone marrow stromal cells (BMSCs) have also been shown to improve functional outcomes in animal models of stroke as well as stroke patients [57]. The mechanism of action has been ascribed to the neurotrophic factors secreted by differentiated BMSCs (e.g., neural, glial, and endothelial cell types). Liu et al. [58] showed that intracerebral BMSC transplantation attenuated the MMP activation and subsequent neurovascular unit destruction caused by tPA treatment (1 h 30 min after MCAO and reperfusion). The authors suggested that the protective effect of BMSCs may be useful for reducing the damage of exogenous tPA in acute thrombolytic therapy for ischemic stroke patients. Considering that MSCs or other types of stem cells may exist endogenously, a better understanding of the therapeutic effects of minocycline or other drugs on both endogenous and exogenous stem cells may optimize such combination therapy.

### 4.2. Normobaric Hyperoxia (NBO) and Hyperbaric Oxygen (HBO) Therapy

NBO treatment affords neuroprotection when initiated early after ischemia onset [59]. Previous studies showed that NBO can protect the BBB against ischemic damage through inhibition of reactive oxygen species (ROS) production and MMP-9 induced damage of TJPs in stroked rats [60]. Early NBO treatment (100% O_2_) was found to attenuate the MMP-9 induction in the ischemic microvessels of tPA-treated rats (tPA given at 3, 5, and 7 h MCAO) [28]. It also prevented the loss of occludin and claudin-5 due to delayed (5 and 7 h MCAO) tPA treatment. Importantly, NBO reduced the HT, brain edema, infarction volume, and mortality in tPA-treated rats. Neurological functions were also improved in rats subjected to NBO plus tPA. It was suggested that NBO could increase tPA’s therapeutic window for ischemic stroke to at least 7 h. Rationally designed clinical studies with well-defined patient populations are required to validate whether NBO is a viable, safe, and efficacious adjunctive treatment for ischemic stroke [28]. Similarly, the documented therapeutic effects of HBO against experimental stroke [61,62] and in the clinic [63,64] warrant studies on its efficacy in combating tPA-induced complications.

### 4.3. Others

Brain imaging has been used to determine patient subgroups with increased risk for hemorrhage and poor clinical outcomes profile [51]. This technique has guided treatment decisions and consequently improved tPA’s therapeutic time window with acceptable safety. Previous trials also demonstrated that endovascular procedures, for example intra-arterial thrombectomy, improved stroke outcomes in patients who received intravenous thrombolysis. In contrast with thrombolysis alone, thrombectomy combined with thrombolysis enhanced functional outcomes and reduced mortality in patients with ischemic stroke [52,53].

## 5. Summary and Conclusions

While we have only included in this review the preclinical studies which specified delayed tPA treatment as >4.5 h post stroke onset, it is noteworthy that some other drugs also attenuated HT and other complications associated with tPA treatment initiated at <4.5 h after stroke (e.g., annexin A2, fingolimod, progesterone, progranulin, uric acid, etc.) in animal models [12,13,14]. Nevertheless, considering that the studies mentioned in this paper are mostly preclinical studies, caution is needed when interpreting the results. As the effects of drug or non-drug interventions were examined in specific groups of animals (i.e., male or female only and/or old or young animals), the influence of age and gender on post-stroke outcomes, specifically delayed tPA-induced HT, needs to be explored. Moreover, rigorous preclinical studies are warranted in view of the clinical finding that erythropoietin, a vascular protective agent, did not reduce but rather increased HT occurrence [65].

Preclinical studies should also focus on interventions that exert neuroprotection in addition to attenuating HT. Notably, a recent meta-analysis involving 6756 participants in the nine clinical trials of intravenous alteplase versus controls showed that the increase in the occurrence of HT has been caused by a number of factors, including stroke severity [66]. Thus, in light of the role that stroke severity plays in HT, interventions given alongside tPA should also exert neuroprotection and accelerate the salvage of brain tissue after stroke.

Because tPA is essential for reperfusion therapy, finding the right dosage and timing of initiating treatment in relation to tPA is important to enhance possible clinical application of the combined therapy. Specifically, it is very difficult to estimate the precise time of stroke onset to administer the drugs in combination with tPA in the clinics. The FDA standards require assurance that any intervention (i.e., drugs) that are given alongside tPA should not block the fibrinolytic activity of tPA [65]. Nevertheless, to identify the appropriate targets and surmise interactions that could enhance the benefits of thrombolytic therapy, it is also imperative that we completely comprehend the exact mechanisms of tPA-induced HT and the other detrimental effects associated with delayed tPA treatment. Moreover, examining long-term efficacy of the combination therapy is also prudent to determine the worth of the drug when given as a treatment to curtail effects of delayed tPA treatment. Long-term efficacy assessments should include examining motor behavior functions not only a few days, but even months after drug treatment in view of the Stroke Treatment Academic Industry Roundtable (STAIR) guidelines [67,68]. In addition, when contemplating combination therapy with stem cells, the Stem cell Therapeutics as an Emerging Paradigm for Stroke (STEPS) recommendation may be helpful in translating these novel therapies to the clinic [69].

Enhancing tPA’s time window via combination therapy will not only significantly improve HT and other side effects associated with delayed tPA therapy, but will also result in enhancement of the risk–benefit ratio for thrombolytic therapy and increase the number of patients eligible for tPA therapy. An expanded treatment window will also allow the time window of neuroplasticity to remain open for a longer period resulting in better recovery and functional outcomes post-treatment. Another potential, significant clinical application of this strategy is the treatment of “wake-up strokes”, a case where patients awaken with stroke symptoms, which poses a significant challenge for acute stroke providers [70]. Combining tPA with interventions that could enhance its therapeutic time window is a reasonable strategy to treat patients with wake-up strokes.

At the time of writing, efforts are still underway to discover other fibrinolytics or thrombolytic drugs with better reperfusion efficacy than tPA [14,71,72]. Nevertheless, it is equally important to also explore logical and effective approaches that could improve the only FDA-approved stroke therapy [9]. As mentioned in this review, combining tPA with drugs and non-drug interventions is one approach that could circumvent the adverse outcomes associated with delayed tPA therapy, and thus, enhance the time window of tPA treatment. 

## Figures and Tables

**Table 1:** Pharmacological adjunctive treatments to extend therapeutic window for ischemic stroke treatment.

Adjunctive Treatment (Dosage, Mode and Timing of Treatment)	Species & Stroke Model	tPA Dose, Mode & Timing of Treatment	Parameter/Molecular Target	Outcome	Timing of Evaluation	Ref.
Ascorbic acid (500 mg, p.o.) 5 h post stroke	Male rats; MCA cauterization	1 mg/kg, i.v., 5 h post stroke	infarct volume	decreased	48 h post stroke	[15]
			brain edema	decreased		
			brain permeability	decreased		
			MMP-9	decreased		
			Sensorimotor functions	improved		
Atovarstatin (First dose: 20 mg/kg 4 h after stroke, Second dose: 20 mg/kg at 24 h after the first dose, s.c.)	Male Wistar rats; embolic	10 mg/kg, i.v., 6 h post stroke	HT		7 h 30 h post stroke	[16]
			infarct volume	decreased		
			neurological functions	improved		
			thrombolysis and vascular patency	increased		
			ICAM-1	reduced		
			PAR-1	reduced		
			Collagen type IV	reduced		
			MMP-9	increased		
Batimastat (MMP inhibitor; 50 mg/kg; i.p., 3 and 6 h after stroke)	Male spontaneously hypertensive rats; embolic	10 mg/kg, i.v., 6 h post stroke	HT	decreased	24 h post stroke	[17]
			infarct volume	decreased		
			neurological functions	improved		
			Mortality	decreased		
Bryostatin (PKC modulator; 2.5 mg/kg, i.v., alongside tPA)	Female SD rats, 18–20 mo old; embolic	5 mg/kg, i.v., 6 h post stroke	HT	decreased	24 h post stroke	[18]
			infarct volume	not changed		
			MMP-9	decreased		
			MMP-2	not changed		
			PKCɛ	increased		
			PKCα	not changed		
			PKCδ	not changed		
Candesartan (AT1R blocker; 1 mg/kg, i.v., 3 h after stroke)	Male Wistar rats (330–350 g); embolic	10 mg/kg, i.v., 6 h post stroke	HT	decreased	24 h post stroke	[19]
			infarct volume	not changed		
			MMP-9	not changed		
			MMP-2	not changed		
			MMP-3	decreased		
			NF-κB	decreased		
			TNF-α	decreased		
			p-eNOS	decreased		
Cilostazol (PDEIII-inhibitor; 10 mg/kg, i.p., before tPA)	Male ddY (22–26 g) 4 weeks old; intraluminal filament/reperfusion	10 mg/kg, i.v., 6 h post stroke, before reperfusion	HT	decreased	18 h post reperfusion 7 days post stroke	[17]
			infarct volume	decreased		
			MMP-9	decreased		
			claudin 5	enhanced		
			locomotor behavior	improved		
Dodecafluoropentane emulsion (DDFPe) nanodroplets 0.3 mL/ kg, i.v. 1 h after stroke, and 5 additional doses at 90 min intervals	New Zealand male or female rabbits; 3.4 to 4.7 kg/bw; Embolic	0.9 mg/kg tPA, 9 h after last DDFPe dose	stroke volume	decreased	24 h post stroke	[20]
			neurological functions	improved		
Fasudil (ROCK inhibitor; 3 mg/kg, i.p., before tPA)	Male SD rats (250–330 g); intraluminal filament/reperfusion	10 mg/kg, i.v., 6 h post stroke, after reperfusion	HT	decreased	18 h post reperfusion 7 days post stroke	[21]
			infarct volume	not changed		
			MMP-9 (in vitro)	decreased		
			locomotor behavior	improved		
G-CSF (300 μg/kg, i.v., alongside tPA)	Male SD rats, (200–250 g) 9–10 weeks old; intraluminal filament/reperfusion	10 mg/kg, i.v., post stroke, before reperfusion	HT	decreased	24 h post drug treatment	[22]
			infarct volume	not changed		
			neurological functions	improved		
			Ang-1	not changed		
			Ang-2	increased		
			CD34	increased		
			eNOS	increased		
			VEGFR2	increased		
			vWF	increased		
GM6001 (MMP inhibitor; 100 mg/kg, i.p., alongside tPA)	Male ddY mice (22–30 g) 4 weeks old; intraluminal filament/reperfusion	10 mg/kg, i.v., 6 h post stroke, after reperfusion	HT	decreased	48 h post stroke/reperfusion	[23]
			infarct volume	not examined		
			MMP-9	decreased		
			claudin (in vitro, in vivo)	not changed		
			occludin (in vitro, in vivo)	enhanced		
			ZO-1 (in vitro, in vivo)	enhanced		
Imatinib (PDGFR-α antagonist; 200 mg/kg, at 1 h after ischemia)	C57BL/6J mice, 10 weeks old, photothrombotic induction of MCAO	10 mg/kg, i.v., 5 h after stroke	HT	decreased	24 h post stroke	[24]
IMM-H004 (Coumarin derivative; 6 mg/kg, i.v., alongside tPA)	Male SD rats (300–320 g); embolic Male SD rats (260–280 g); intraluminal filament/reperfusion	10 mg/kg, i.v., post stroke	HT	decreased	18 h post stroke 24 h post stroke 1, 2, 3 days post stroke 24 h post stroke 1–7 days post stroke 24 h post stroke/reperfusion 7 days post stroke/reperfusion	[25]
			infarct volume	decreased		
			neurological functions	improved		
			HT	decreased		
			infarct volume	decreased		
			neurological functions	improved		
			pro-MMP-9	decreased		
			Akt (in vitro)	decreased		
			Ang-1	increased		
			CD31	increased		
			CD31 + Ki67	increased		
			MMP-2	not co-localized in astrocytes		
			occludin	decreased		
			Tie2	increased		
Minocycline (antibiotic; 3 mg/kg, intravenous (i.v.), 4 h after stroke)	Male SHR; embolic	10 mg/kg, i.v., 6 h post stroke	HT	decreased	24 h post stroke	[26]
			infarct volume	decreased		
			MMP-9 (plasma)	decreased		

Abbreviations: tPA, tissue plasminogen activator; SHR, spontaneously hypertensive rat; HT, hemorrhagic transformation; PDEIII, phosphodiesterase III; MMP, matrix metalloproteinase; ZO, zonula occludens; ROCK, Rho-associated protein kinase; SD, Sprague Dawley; AT1R, angiotensin II type 1 receptor; MCAO, middle cerebral artery occlusion; NF-κB, nuclear factor NF-κB; TNF-α, tumor necrosis factor; eNOS, endothelial nitric oxide synthase; ICAM-1, Intercellular Adhesion Molecule 1; PAR-1, Protease-activated receptor-1; PKC, protein kinase C, Akt or protein kinase B, Ang, angiotensin, CD, cluster of differentiation; Tie, tyrosine kinase with Ig and EGF, G-CSF, granulocyte-colony stimulating factor; VEGFR2, vascular endothelial growth factor receptor 2; vWF, Von Willebrand factor.

**Table 2:** Non-drug adjunctive treatments to extend therapeutic window for ischemic stroke treatment.

Adjunctive Treatment (Dosage, Mode and Timing of Treatment)	Species & Stroke Model	tPA Dose, Mode & Timing of Treatment	Parameter/Molecular Target	Outcome	Timing of Evaluation	Ref.
Neural stem cells (1 day post stroke) + minocycline	Aged mice	10 mg/kg, i.v., 6 h post stroke	neurological functions	improved	48 h post stroke	[27]
	Intraluminal filament model		mortality	reduced		
Normobaric oxygen (100% O_2_)	Male Sprague-Dawley rats (290–320 g) suture occlusion, and reperfusion	10 mg/kg, i.v., 5 and 7 h post stroke, 15 min prior to reperfusion	HT	reduced	24 h post stroke	[28]
			infarct volume	reduced		
			brain edema	reduced		
			BBB disruption	reduced		
			MMP-9	reduced		
			Occludin	enhanced		
			Claudin-5	enhanced		
			neurological deficits	reduced		
			mortality	decreased		

Abbreviations: BBB, blood-brain barrier; HT, hemorrhagic transformation; MMP, matrix metalloproteinase.
